# Nutritional Status of Patients with Cancer During Oncological Treatment—A Multicenter Cross-Sectional Study

**DOI:** 10.3390/nu18172901

**Published:** 2026-09-04

**Authors:** Anna Lewandowska, Tomasz Lewandowski, Michał Próchnicki, Aleksandra Stryjkowska-Góra, Tomasz Góra, Grzegorz Rudzki, Barbara Laskowska, Barbara Stawarz, Dorota Durda, Beata Piwińska, Beata Jurek, Iwona Maziarek

**Affiliations:** 1 Faculty of Healthcare, State Academy of Applied Sciences in Jaroslaw, Czarniecki Street 16, 37-500 Jarosław, Poland; 2 Faculty of Medical and Health Sciences, State Vocational University in Tarnobrzeg, Henryk Sienkiewicz Street 50, 39-400 Tarnobrzeg, Poland; 3 Faculty of Technical Engineering, State Academy of Applied Sciences in Jaroslaw, Czarniecki Street 16, 37-500 Jarosław, Poland; 4 I Department of Psychiatry, Psychotherapy and Early Intervention, Medical University of Lublin, Głuska 1 Street, 20-439 Lublin, Poland; 5 Department of Oncology, Radiotherapy and Translational Medicine, University of Rzeszow, Glin. Tadeusza Rejtana 16C, 35-959 Rzeszow, Poland; 6 Clinical Department of Gynecology and Obstetrics, Independent Public Health Care Complex in Rzeszów, Rycerska Street 4, 35-055 Rzeszow, Poland; 7 Department of Endocrinology, Medical University of Lublin, Jaczewskiego Street 8, 20-439 Lublin, Poland; 8 Collegium Masoviense, University of Health Sciences in Zyrardow, Narutowicza Street 35, 96-300 Zyrardow, Poland

**Keywords:** nutrition assessment, malnutrition, risk factors, cancer

## Abstract

**Background**: Malnutrition is a significant clinical problem in patients with cancer, occurring not only during hospitalization but also throughout the recovery phase. It brings severe health and economic consequences, affecting patients on both physical and psychological levels. The American Society for Parenteral and Enteral Nutrition (ASPEN) and the European Society for Clinical Nutrition and Metabolism (ESPEN) emphasize the critical importance of early detection of nutritional disorders and timely intervention in oncology populations. The aim of this study was to determine the prevalence of malnutrition and factors associated with malnutrition among hospitalized Polish oncology patients and to draw attention to the need for early nutritional assessment in patients with cancer so as to ensure appropriate nutritional care. **Methods**: A multicenter cross-sectional study was conducted in accordance with the Strengthening the Reporting of Observational Studies in Epidemiology (STROBE) guidelines among adult patients diagnosed with cancer and undergoing oncological treatment. Data collection involved a structured survey questionnaire, anthropometric measurements, and validated screening tools: Mini Nutritional Assessment (MNA), Nutritional Risk Screening Score 2002 (NRS 2002), and Subjective Global Assessment (SGA). **Results**: The study group comprised 560 patients with a mean age of 55 ± 13.95 years, including 59% women and 41% men. The mean duration of illness was 3.37 years. Advanced-stage cancer was present in 43% of participants, and 66% were undergoing chemotherapy. The mean Body Mass Index (BMI) for the study group was 23.14 kg/m^2^, with a mean MNA score of 16.23 points and a mean NRS 2002 score of 2.39 points. Based on Subjective Global Assessment, moderate malnutrition was identified in 45% of patients, whereas severe malnutrition was confirmed in 27% of the study population. **Conclusions**: The prevalence of malnutrition among oncology patients is high. Early screening and regular monitoring are essential for the prompt identification of patients at risk, enabling the implementation of appropriate nutritional therapy and the improvement of clinical outcomes.

## 1. Introduction

According to data published by the International Agency for Research on Cancer (IARC), approximately 20 million new cancer cases and 9.7 million cancer-related deaths were recorded worldwide in 2022. Data collected by the Global Cancer Observatory (GCO) indicate that the number of new cancer cases is expected to exceed 35 million by 2050 [1]. According to the report of the Polish National Cancer Registry, malignant neoplasms constitute an increasing health, social, and economic burden in Poland. The scale of this problem is reflected by approximately 170,000 new cancer diagnoses and more than 100,000 cancer-related deaths annually. Currently, over 1.17 million Poles are living with cancer. Cancer incidence increases exponentially with age, rising approximately tenfold every two to three decades of life [2].

Malnutrition represents a major problem in patients with cancer. According to the World Health Organization (WHO), malnutrition affects every country worldwide and occurs across all age groups, regardless of sex or socioeconomic status [3]. Symptoms of malnutrition or cachexia are present in 36% of oncology patients and may affect 50–80% of individuals with advanced-stage disease. According to data published by the European Society for Clinical Nutrition and Metabolism (ESPEN), nutritional status deteriorates significantly during hospitalization in 30% of previously well-nourished patients and in as many as 70% of patients with pre-existing malnutrition. Furthermore, 20% of oncology patients die directly as a consequence of malnutrition [4,5,6,7]. The Polish Supreme Audit Office reports that the quality of oncology care in Poland remains lower than in most European Union countries and that malnutrition affects between 30% and 85% of Polish oncology patients, most commonly in those with advanced stage of the disease [3,8]. The most recent report of the Polish Society for Parenteral, Enteral Nutrition and Metabolism estimates that at least 30% of patients hospitalized in Poland are malnourished [9].

Malnutrition is recognized as a clinical entity and is included in the International Statistical Classification of Diseases and Related Health Problems (ICD-10). ESPEN defines malnutrition as a state resulting from a lack of absorption or intake of nutrients, leading to altered body composition and impaired physical and psychological function, which adversely affects the clinical outcome of the primary disease. The World Health Organization (WHO) defines malnutrition as an imbalance between the supply of nutrients and energy and the body’s requirements for optimal functioning [7,10].

The etiology of malnutrition in oncology patients is complex and multifactorial. Nutritional deficiencies can be observed at every stage of the neoplastic process, arising as a result of the therapy itself or manifesting during the terminal phase of the disease. The severity of these disorders depends on the tumor type and location, the stage of disease progression, the patient’s age, and the treatment modalities used [11]. Malnutrition in oncology patients results from insufficient nutrient intake, which can lead to the depletion of body fat and lean mass, ultimately reducing physical performance. Initially, cancer patients may experience anorexia due to altered appetite-regulating signaling. Patients may also face physical limitations that reduce food intake and nutrient absorption, such as stomatitis, diarrhea, vomiting, pain, bowel obstruction, or malabsorption. Systemic inflammation—driven by the release of pro-inflammatory cytokines from the tumor or immune cells—is common in cancer. This state can increase metabolic demands, suppress appetite, and initiate accelerated muscle protein catabolism [10,11,12].

Malnutrition is a significant problem not only during hospitalization but also throughout the recovery phase, contributing to serious health and economic consequences on both physical and psychological levels. Research indicates that malnutrition is associated with poorer response and tolerance to anticancer treatment, reduced quality of life, and higher rates of morbidity, mortality, and infectious complications. Furthermore, weight loss can lead to increased chemotherapy toxicity. A correlation has also been demonstrated between malnutrition and prolonged hospital stays, as well as a significant increase in the costs of treatment and care [10,11].

Despite the high prevalence of oncological malnutrition and the awareness of its contribution to increased mortality, it remains frequently underdiagnosed. The American Society for Parenteral and Enteral Nutrition (ASPEN) and the European Society for Clinical Nutrition and Metabolism (ESPEN) emphasize the importance of early detection of nutritional disorders and intervention in oncology populations. Therefore, diagnosing malnutrition, identifying factors associated with nutritional disorders upon hospital admission, and ensuring the proper nutritional status of cancer patients are vital components of the treatment process and early nutritional intervention to prevent further complications [5,11].

In Poland, the Ministry of Health introduced a mandatory nutritional assessment procedure for all hospitalized patients on 1 January 2012, applicable to all hospital departments except emergency departments. The assessment is required to be performed at admission using one of two recommended screening tools: the NRS 2002 Nutritional Risk Screening or the SGA Subjective Global Assessment. Unfortunately, compliance with this requirement remains insufficient, and malnutrition, despite being a serious clinical problem, continues to be underestimated. Nutritional screening is often performed too late, frequently at the time of discharge, and the results are not consistently used as a basis for implementing nutritional therapy. The most recent nationwide survey, conducted in 2018, demonstrated that nutritional interventions implemented in Polish hospitals were inadequate [3,9].

Given that these findings are no longer current, the present study sought to determine the prevalence of malnutrition and the factors associated with malnutrition among hospitalized patients with cancer. Indirectly, the study aimed to increase the importance of early nutritional assessment in oncology patients and the need to provide adequate nutritional care. To achieve this objective, both nationally recommended screening tools, Nutritional Risk Screening Score 2002 and Subjective Global Assessment, were applied. In addition, anthropometric measurements, as the simplest indicators of nutritional status, and the Mini Nutritional Assessment (MNA) were used to evaluate and compare these instruments in identifying patients at risk of malnutrition.

## 2. Data and Methods

### 2.1. Study Design

A multicenter cross-sectional study was conducted in 2025 in accordance with the Strengthening the Reporting of Observational Studies in Epidemiology (STROBE) guidelines among adult patients diagnosed with cancer and undergoing oncological treatment. The study group included patients diagnosed with histopathologically confirmed lung cancer, breast cancer, colorectal cancer, bladder cancer, and head and neck cancer. The study was carried out in oncology departments located in the Podkarpackie Voivodeship, including the Specialist Hospital in Brzozów, the University Hospital in Rzeszów, the Provincial Hospital in Przemyśl, and the Medical Care Center in Jarosław.

Patients underwent nutritional assessment within three days of hospital admission. A stratified random sampling strategy was applied based on the following inclusion criteria: age ≥ 18 years, provision of informed consent, confirmed diagnosis of cancer, ongoing oncological treatment for at least 6 months, and a health status enabling participation in the study. Exclusion criteria included refusal to participate, age below 18 years, cognitive impairment preventing meaningful communication, a clinical condition compromising patient safety, and an oncological treatment duration of less than 6 months. The required sample size was calculated using the StatCalc function of Epi-Info 3.5.3. Assuming a prevalence of malnutrition among oncology patients of 30%, a significance level of 5%, statistical power of 80%, and a relative precision of 20%, a minimum sample size of 224 participants was required. Patient recruitment was performed by a member of the research team at each participating center and consisted of direct selection of participants from the inpatient population of a given oncology department. The patient population within each department was divided into homogeneous strata representing specific cancer groups. Subsequently, independent stratified random samples were drawn within each stratum to ensure optimal sample selection in accordance with the predefined study criteria [13]. Data collection was conducted by trained members of the research team using predefined criteria for the diagnosis of malnutrition. Before initiating the main study at each participating center, a pilot study involving five participants was conducted to verify the questionnaire and assess whether all questions were clear and understandable, interpreted according to the investigator’s intentions, and capable of generating the information sought by the researchers. No concerns or objections were raised by participants during the pilot phase. Data obtained during the pilot study were not included in the final statistical analysis. Once the pilot phase was complete, the main study was initiated. Prior to enrollment, each patient received detailed information regarding the purpose and significance of the study and was informed that participation was entirely voluntary. Study information sheets and informed consent forms were distributed to all eligible participants. Taking into account refusals and withdrawals, 150 patients were recruited into each cancer group. Only fully completed questionnaires were included in the statistical analyses, whereas incomplete questionnaires were excluded.

### 2.2. Criteria for the Diagnosis of Malnutrition

Assessment tools were used that allowed the evaluation of nutritional risk from the patient’s subjective perspective as well as an objective measurement of nutritional status. In addition, biometric, anthropometric, and social variables were used as factors determining health.

A proprietary interview questionnaire was used, which contained questions regarding demographic data, cancer disease, treatment received, method of nutrition, and assessment of the presence of etiological abnormalities. It allowed the identification of insufficient food intake caused by diseases of the oral cavity, swallowing difficulties, reduced appetite, taste disturbances, or gastrointestinal symptoms. In the assessment of the method of nutrition, a distinction was made between “natural nutrition”, defined as enteral intake through the consumption of nutrients directly into the gastrointestinal tract by means of an oral diet; “artificial nutrition”, defined as the administration of specialized nutritional preparations or industrial diets through an artificial access to the gastrointestinal tract, such as a feeding tube or gastrostomy (PEG); and “parenteral nutrition”, defined as the administration of nutrients directly into the bloodstream, bypassing the gastrointestinal tract.

The main phenotypic criteria used in the assessment were body mass index (BMI), unintentional weight loss, and reduced muscle mass.

Body mass index was calculated as the ratio of body weight to the square of height. Based on the criteria established by the World Health Organization (WHO), BMI was classified into the following categories: underweight (<18.5 kg/m^2^), normal body weight (18.5–24.9 kg/m^2^), overweight (25.0–29.9 kg/m^2^), and obesity (≥30.0 kg/m^2^) [14].

In the course of cancer, unintentional weight loss of more than 5% within 6 months is an indicator of malnutrition or the risk of malnutrition [15].

Mid-arm circumference (MAC) measurement is a rapid anthropometric method used to assess nutritional status and muscle mass, measured at the midpoint between the acromion and olecranon processes. Reference values are 16–23 cm for women and 18–25 cm for men, and a reduction in this measurement may indicate malnutrition [15].

Nutritional status was assessed using Polish versions of nutritional screening scales. These instruments had been previously validated and published in the Polish scientific literature.

The Mini Nutritional Assessment (MNA) is a recognized screening tool used to assess malnutrition in older adults, taking into account decreased appetite, weight loss, mobility, psychological stress, neuropsychological problems, BMI, and calf circumference. A score of 24–30 points indicates normal nutritional status, 17–23.5 points indicates a risk of malnutrition, and 0–16.5 points indicates malnutrition [15,16].

The Nutritional Risk Screening 2002 (NRS 2002) is a validated screening tool used to assess the risk of malnutrition in hospitalized patients, based on nutritional status and disease severity. A score of 3 points or more may constitute an indication for nutritional therapy [15,16].

The Subjective Global Assessment (SGA) scale is a method of subjective assessment of a patient’s nutritional status, based on medical history (changes in body weight, diet, gastrointestinal symptoms) and physical examination (loss of adipose tissue, loss of muscle mass, edema). The result classifies the patient into one of three categories: A (well nourished), and B (moderate malnutrition/suspected malnutrition), or C (severe malnutrition). Categories B and C in the SGA questionnaire may constitute an indication for nutritional therapy [15,16].

### 2.3. Sample

The study included 560 patients, 59% were women and 41% were men. The mean age of the participants was 55.0 ± 13.95 years. For analytical purposes, patients were categorized into five cancer groups according to tumor location. Group I consisted of patients diagnosed with breast cancer, Group II included patients with colorectal cancer, Group III comprised patients with bladder cancer, Group IV included patients with lung cancer, and Group V consisted of patients diagnosed with head and neck cancer.

### 2.4. Ethical Considerations

The study was conducted in accordance with the Declaration of Helsinki, and the protocol was approved by the Team for Scientific Research Ethics at the Bioethics Committee of the State Academy of Applied Sciences in Tarnobrzeg (No. 8/2024/2025 dated 15 January 2025; study performed at the Specialist Hospital in Brzozów) and the Bioethics Committee at Collegium Masoviense University of Health Sciences in Zyrardow (No. 5054/2024; study performed at the Provincial Hospital in Przemyśl, 5130/2024; study performed at the Medical Care Center in Jarosław, 5429/2025; study performed in the Podkarpackie Voivodeship, including the University Hospital in Rzeszów). Participation in the study was voluntary and anonymous and respondents were informed of their right to refuse or withdraw from the study at any time. Each participant was informed of the study’s purpose and duration. All patients provided written informed consent to participate in the study before being enrolled in the study.

### 2.5. Data Analysis

The analysis was performed using the IBM SPSS 26.0 software package together with the ExactTests module. Continuous variables were presented as means and standard deviations (SD), whereas categorical variables were presented as frequencies and percentages. The Chi-square (χ^2^) test of independence was used to examine the existence of associations between selected variables. A *p*-value of <0.05 was considered statistically significant.

## 3. Results

### 3.1. Demographics

Group I comprised patients with breast cancer, Group II patients with colorectal cancer, Group III patients with bladder cancer, Group IV patients with lung cancer, and Group V patients with head and neck cancer. The study group had a mean age of 55 ± 13.95 years, with the youngest participant being 19 years old and the oldest 91 years old. Women accounted for 59% of the total cohort. The patients were predominantly rural residents (51%). A good financial situation was reported by 37% of all patients, 32% had secondary education, 32% were professionally active, and more than half were married (56%). Detailed descriptive statistics characterizing the study group are presented in Table 1.

### 3.2. Cancer History

The mean duration of illness was 3.37 years (SD = 2.39). More than half of the study participants (60%) had been undergoing cancer treatment for more than one year, with overall disease duration ranging from 1 to 14 years. Advanced-stage cancer was present in 43% of the participants, and 66% were currently receiving chemotherapy (Table 2). The vast majority of the patients (88%) received family support during their illness. Regarding self-assessed health status, 41% rated their health as poor, 25% as fair, and only 14% as good, while 20% were unable to provide a definitive assessment.

**Figure 1:**
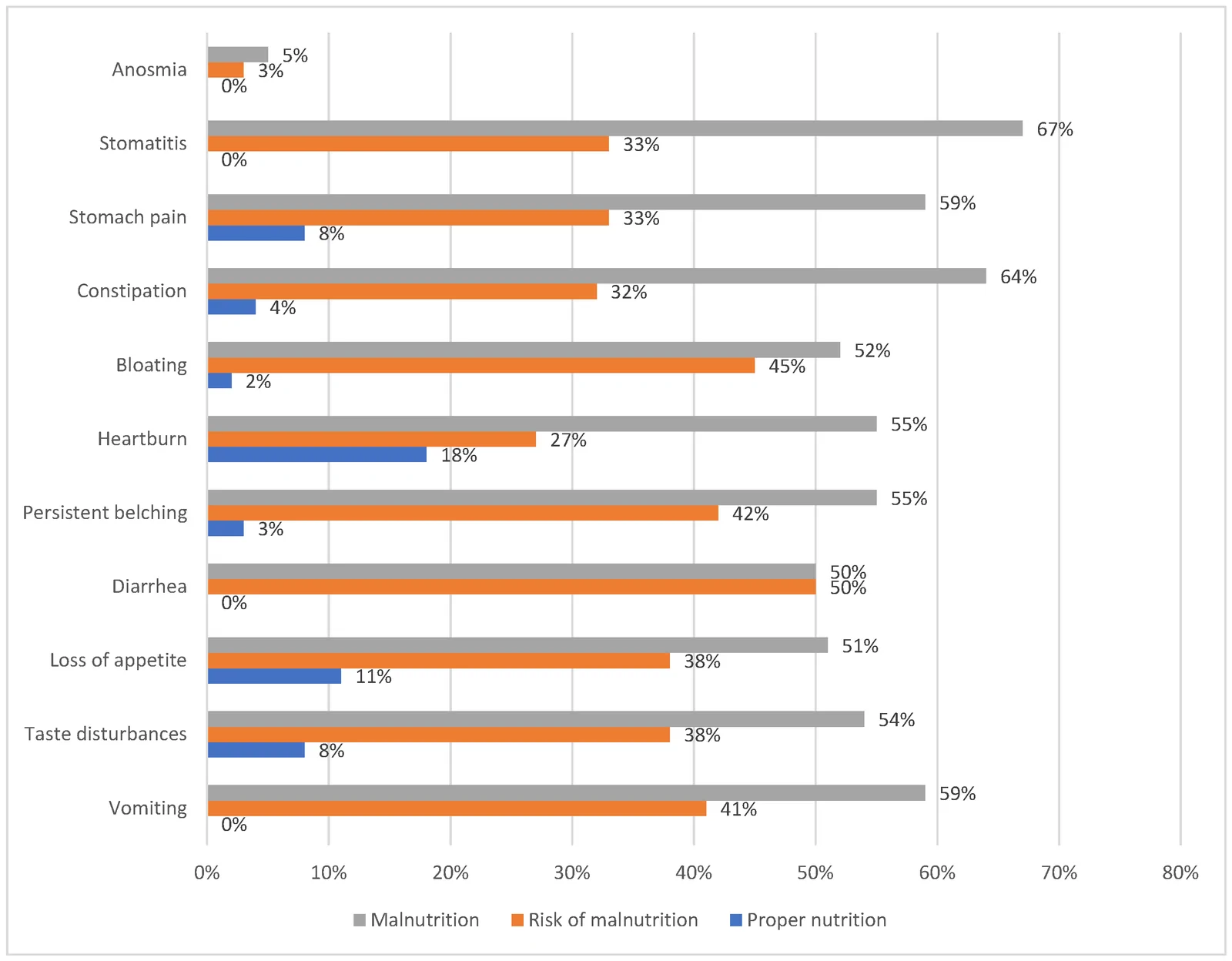
The impact of ailments on the occurrence of malnutrition.

**Figure 2:**
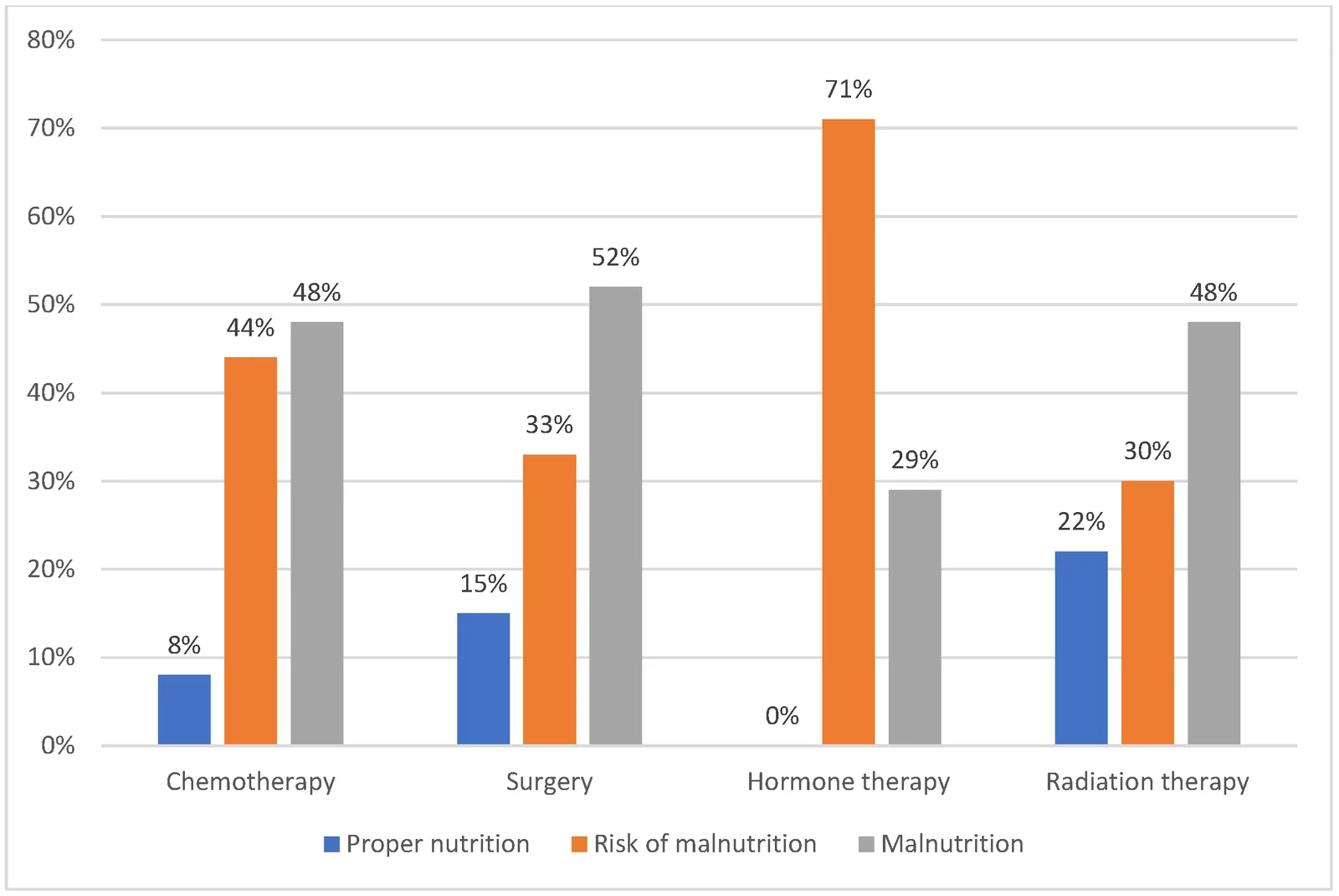
The occurrence of malnutrition depending on the oncological therapy used.

The most frequently reported symptoms among malnourished patients were stomatitis (67%) and constipation (64%). More than half of the study participants (67%) who experienced distressing symptoms related either to the underlying malignancy or to oncological treatment were malnourished. The prevalence of malnutrition was significantly higher among patients presenting with gastrointestinal symptoms (*p* < 0.001) (Figure 1). The weakest association between treatment modality and the occurrence of malnutrition was observed for hormonal therapy (Figure 2).

The vast majority of patients were receiving nutrition orally (83%) (Table 2). Among the patients, 35% were under the care of a nutritional outpatient clinic, and 26% remained under the supervision of a dietitian.

### 3.3. Nutritional Status

The phenotypic criteria applied in the assessment of nutritional status were body mass index (BMI), reduced muscle mass determined by mid-arm circumference (MAC), and unintentional weight loss. The mean BMI for the study group was 23.14 (SD = 4.37), ranging from 13.84 to 46.06 kg/m^2^. Underweight was more frequently indicated by the BMI of participants receiving natural nutrition (14%) compared to those requiring artificial nutritional support (9%).

Patients with normal body weight were most frequently observed among patients with intermediate-stage disease. The χ^2^ test did not confirm the existence of a statistically significant association between these variables at the adopted level of significance (*p* = 0.140). The study demonstrated a correlation between tumor location and BMI value. More than half of the participants (60%) with head and neck cancer obtained a result indicating underweight (*p* = 0.001) (Table 3 and Table 4).

Unintentional weight loss, defined as a loss of more than 5% of body weight in 6 months, is another indicator of malnutrition or the risk of malnutrition. Actual weight loss ranged from 1 to 20 kg. The prevalence of unintentional weight loss was higher among patients with lung cancer compared with patients with other cancer locations (*p* < 0.001) (Table 3).

Mid-arm circumference (MAC) values below the sex-specific cutoff thresholds (<16 cm for women and <18 cm for men), indicating malnutrition, were observed in 28% of all patients. This was significantly more frequent among patients with head and neck cancer compared to those with other cancer sites (*p* < 0.001) (Table 3).

In the MNA scale, patients scored an average of 16.23 points (SD = 4.97), with scores ranging from 3 to 27.5 points. Nutritional status assessment using the Mini Nutritional Assessment (MNA) revealed that malnutrition was most prevalent among male patients, individuals with secondary education, rural residents, and those reporting average or poor socioeconomic status. Malnutrition was most frequently identified among patients with lung cancer (91%), whereas the lowest prevalence was observed in patients with breast cancer (14%). The highest proportion of patients with normal nutritional status (58%) was found among patients who had been undergoing treatment for more than five years, while the lowest proportion (2%) was observed among those with the shortest treatment duration. Among patients with advanced-stage cancer, as many as 83% were classified as malnourished. Furthermore, malnutrition occurred more frequently among patients receiving artificial nutritional support (64%) than among those fed through the natural route (Table 5).

In the NRS scale, the mean score obtained by patients was 2.39 (SD = 1.31), ranging from 0 to 5 points. Indications for nutritional therapy were more frequent among men (59% of men vs. 41% of women), rural residents (56% rural vs. 44% urban). Among study participants, patients with head and neck cancer (80%) were more likely to meet the criteria for nutritional therapy according to the NRS 2002. However, the chi-square test did not confirm a statistically significant association between cancer type and the indication for nutritional treatment. During the first year of oncological therapy, 45% of patients met the criteria indicating the need for nutritional intervention. In contrast, among patients who had been receiving treatment for 4 to 5 years, the proportion requiring nutritional support increased to 80% (Table 6).

Based on the Subjective Global Assessment (SGA), moderate malnutrition was identified in 45% of all patients, whereas severe malnutrition was confirmed in 27% of the study population. Subjective assessment of nutritional status was not associated with age, place of residence, or socioeconomic status (*p* = 0.2025). The risk of malnutrition was more frequently observed among male patients (53%), whereas severe malnutrition was more common among female patients (38%). Severe malnutrition (54%) was observed more frequently among patients with a cancer duration exceeding four years (*p* = 0.001). Severe malnutrition (42%) was most frequently identified among patients with lung cancer (*p* = 0.01) (Table 7).

## 4. Discussion

Hospital-based studies in Europe have shown that the prevalence of malnutrition among oncology patients ranges from 25% to over 70% based on nutritional status assessments, and cancer patients are among the most malnourished patient groups [17,18,19,20,21]. In patients with cancer, the risk of developing malnutrition is influenced mainly by the type of anticancer treatment, drug–food interactions, and nutrition-related complications arising from the selected treatment modality. These include appetite loss, changes in taste and smell perception, rapid weight loss, oral pain, nausea, vomiting, diarrhea, as well as systemic inflammation and infections [3,5,16,17].

Unfortunately, disease-related malnutrition remains an underestimated and frequently neglected clinical problem in Poland. This is reflected by its high prevalence, the absence of a comprehensive nutritional strategy within the National Health Program, the lack of standardized hospital nutrition protocols, limited interest in clinical nutrition among healthcare institutions, insufficient employment of dietitians in hospitals, and the inadequate implementation of nutritional therapy. This situation persists despite the fact that the adverse consequences of malnutrition have been extensively documented. It is also well established that a patient’s nutritional status has a substantial impact on the effectiveness of anticancer treatment. The nutritional status of hospitalized patients in Poland is well illustrated by the results of the nutrition screening survey. According to the most recent report, only three of the ten analyzed hospital wards had access to a dietitian. During hospitalization, malnutrition monitoring was performed primarily through visual assessment or by measuring body weight and calculating BMI. Moreover, body weight was usually assessed only at admission or during the first 24 h of hospitalization. In more than 80% of patients, neither nutritional risk nor malnutrition was formally identified. The vast majority of patients received a standard hospital diet, whereas fewer than 12% received an enriched hospital diet. Specialized diets were prescribed to only 8% of patients, a rate approximately four times lower than that reported in other countries. Despite this, 52% of patients did not consume the entire hospital meal provided to them. The most frequently reported reasons were loss of appetite and dissatisfaction with the taste of the meals. Although caloric requirements were estimated for more than half of hospitalized patients, the calculation of protein requirements, maintenance of detailed nutrition records, and consultations with qualified nutrition professionals were rare practices. Such interventions were implemented significantly more frequently in other countries. Unintentional weight loss during the previous three months was reported by 42% of patients, while only one in five patients received nutritional support. Furthermore, patients in other countries reported substantially higher satisfaction with hospital meals than patients treated in Poland [3,22].

Therefore, comprehensive measures aimed at improving the nutritional status of oncology patients are urgently needed. Such actions can enhance the quality and effectiveness of anticancer treatment in Poland while simultaneously reducing healthcare costs associated with cancer care [3]. Consequently, for several years, published expert guidelines [23,24] and the European Society for Clinical Nutrition and Metabolism (ESPEN) have recommended using the Global Leadership Initiative on Malnutrition (GLIM) algorithm for diagnosing malnutrition, along with nutritional screening and assessment for all oncology patients. These should utilize not only conventional anthropometric measurements but, primarily, screening tools and methods that account for risk factors [25,26,27].

In the present study, several nutritional assessment scales were used to verify the sensitivity of these instruments in assessing the risk of malnutrition and to provide reliable screening of individuals at risk of malnutrition. According to the findings of our study, 20% of patients were classified as underweight based on phenotypic assessment. The lowest BMI value was 13.84 kg/m^2^, whereas the highest was 46.06 kg/m^2^. These findings reflect the limitations associated with the use of body mass index alone for establishing cut-off points for the risk of malnutrition. This further emphasizes that oncology patients, including those with normal body weight and those who are overweight, may be at risk of malnutrition.

According to the Mini Nutritional Assessment (MNA), 54% of patients were malnourished. Based on the Nutritional Risk Screening 2002 (NRS 2002), 56% of patients required nutritional therapy. In the Subjective Global Assessment (SGA), severe malnutrition was identified in 27% of patients. The high prevalence of malnutrition found in our study is not surprising, given that hospitalized oncology patients are more likely to experience some degree of nutritional impairment compared to other hospitalized patients. This is also confirmed by other studies. Silva et al., using the Subjective Global Assessment (SGA) in a cohort of 277 patients, demonstrated that the prevalence of malnutrition was 71.1%, with similar frequencies observed for moderate malnutrition (35.4%) and severe malnutrition (35.7%). Regarding cancer site, severe malnutrition was more most prevalent among patients with upper gastrointestinal tract cancers (28.3%). Among the 126 patients who recalled their usual body weight, 80.2% had experienced a percentage weight loss (%WL) ≥ 5% during the previous 6 months, with a median weight loss of 14.7%, whereas among patients with tumors classified as “other”, 24.8% had a %WL ≥ 5% during the previous 6 months [11]. In the epidemiological, cross-sectional, multicenter study conducted by Waitzberg et al., which included 4000 hospitalized patients aged 18 years or older receiving care within the Brazilian public healthcare system, malnutrition was identified in 48.1% of patients, while severe malnutrition was present in 12.5% [28]. Similarly, Correia et al., in a cross-sectional multicenter epidemiological study involving 9348 hospitalized patients aged over 18 years in Latin America, reported malnutrition in 50.2% of participants, whereas severe malnutrition was observed in 11.2% of the total study population [29]. Based on MNA scores, the PreMio study—a prospective, observational, multicenter study conducted in 22 oncology centers across Italy—showed that among 1952 patients, 51% had nutritional disorders, 9% were overtly malnourished, and 43% were at risk of malnutrition [10]. Similar results were obtained in studies in France, where the prevalence of malnourished patients was 39% [19], and in studies by de la Torre-Montero et al., where malnutrition risk was identified in 38.6% of patients according to NUTRISCORE and 20.5% according to the PG-SGA scale [30].

According to the Global Leadership Initiative on Malnutrition (GLIM) criteria, the diagnosis of malnutrition requires at least one phenotypic criterion and at least one etiologic criterion. Multiple risk factors are associated with the development of malnutrition. A characteristic prognostic indicator in oncology patients is the presence of nutrition-impact symptoms (NIS), due to their high prevalence resulting from the tumor itself, the anticancer treatment administered, and malnutrition as a condition [11]. Our results demonstrated that 53% of the participating patients experienced anorexia (loss of appetite) associated with their neoplastic disease and treatment. Nearly half of the patients also reported abdominal pain (45%), vomiting (41%), and constipation (41%). Silva et al., in a study conducted among 277 Brazilian oncology patients, demonstrated that more than half of the participants (67.1%) experienced nutrition-impact symptoms, the most common of which were anorexia (50.5%), pain (23.1%), vomiting (19.4%), constipation, and taste disturbances (17.7%) [11]. These findings are consistent with other cross-sectional studies that rank anorexia and pain among the most frequent symptoms in their populations [31,32]. A prospective study conducted in Canada on patients with advanced cancer showed that malnutrition symptoms (anorexia, early satiety, dysgeusia, xerostomia, and dysphagia) were associated with reduced survival in univariate analysis, with dysphagia remaining an independent prognostic factor in the final model. This fact highlights the importance of assessing and appropriately managing these symptoms, given their significant impact on prognosis and the patients’ quality of life [33].

Kłęk et al. noted that the risk of malnutrition increases with age and is twice as high in patients over 70 years of age compared to those who have reached 60 years of age [26,27]. According to Silva et al., older age is a factor that increases the risk of malnutrition by nearly 30% [11]. A correlation between malnutrition and age was also demonstrated by Waitzberg et al. (60 years, *p* < 0.05) [28], as well as Correia et al. (>60 years, *p* < 0.05) [29]. In our own research, malnutrition in oncology patients was correlated with their age; more than half of the patients (53%) over 71 years of age were malnourished, whereas in the group under 50 years old, this rate was approximately 44%.

It is well-established in the scientific community that the type of cancer and the treatment modality are predictive factors for malnutrition. Based on recent statistical data, patients with gastrointestinal or head and neck cancers are at a higher risk of malnutrition [1,34,35]. In the present study, malnutrition was most frequently observed among patients with lung cancer and head and neck cancer, whereas the lowest prevalence was found among patients with breast cancer. Similarly, Kłęk et al. report that the risk of malnutrition is particularly high in patients with gastrointestinal and head and neck cancers. Reasons for malnutrition include insufficient oral nutrient intake, loss of appetite, side effects of oncological treatment, and tumor location that hinders eating, chewing, or swallowing [26,27]. In the study by Muscaritoli et al., patients with the highest prevalence of malnutrition were those with gastric, esophageal, pancreatic, head and neck, and lung tumors, while breast cancer patients were the least affected [10]. Trabulo et al. recorded the highest prevalence of nutritional risk in patients with colorectal cancer (25%), followed by breast cancer (21%) and other gastrointestinal cancers (14%). However, analyzing the percentage change over the last six months, they found that gastrointestinal and colorectal cancers were characterized by a higher prevalence of malnutrition than breast cancer [36]. In the study by Marshall et al., patients with upper gastrointestinal, head and neck, and lung cancers were more susceptible to malnutrition [37]. This is confirmed by recent Spanish reports, where the highest percentage of patients at risk of malnutrition were those with pancreatic cancer (89.5%), followed by colorectal (55.3%) and lung cancer (29.3%). Interestingly, the percentage of patients at risk of malnutrition was significantly higher in the group treated with combination therapy (chemotherapy and/or radiotherapy) than in the group treated with immunotherapy alone (58.5% compared to 28.3%, respectively, *p* < 0.0001) [30]. A study by El-Najjar et al. showed that the majority (80.3%) of patients included in the study were malnourished after completing a chemotherapy regimen, with 41.0% being severely malnourished and 39.3% being moderately malnourished or suspected of malnutrition. For comparison, 36.0% of patients were malnourished at baseline, before the first cycle of chemotherapy, with 15.6% being severely malnourished and 20.3% being moderately malnourished or suspected of malnutrition. The difference in SGA category proportions between baseline and post-chemotherapy was statistically significant (*p* < 0.001) [38]. In a one-day survey conducted across 154 hospital wards in 24 French cities, malnutrition was identified in 67% of patients with pancreatic cancer, 60% of those with gastric and/or esophageal cancer, 52% of patients with kidney or bladder cancer, 49% of those with head and neck cancer, 45% of patients with lung cancer, and 41% of patients with ovarian or cervical cancer. The study also demonstrated that malnutrition was more prevalent among patients with metastatic disease, corresponding to advanced-stage cancer. Nutritional support was provided to 57.6% of malnourished patients and to 28.4% of patients with normal nutritional status. The main cause of malnutrition was a reduced amount of food intake, which resulted primarily from decreased appetite (63%) and taste disturbances (42%) [19].

Patients with cancer, particularly those undergoing chemotherapy, are at increased risk of malnutrition. The findings of the present study, together with those reported by other authors, further confirm the persistent under-recognition of malnutrition as a public health problem. This is evidenced by the fact that the prevalence of malnutrition has not decreased despite extensive documentation over recent decades and its well-established association with prognosis and quality of life in patients with cancer.

Nutritional screening and comprehensive nutritional assessment constitute essential components of the management of every patient with cancer. Such assessments should be performed at the beginning of treatment, throughout the course of therapy, and following completion of chemotherapy in order to identify patients who are malnourished or at risk of malnutrition. Nutritional assessment should include a detailed dietary history, evaluation of nutritional disorders and unintentional weight loss during the previous six months, as well as the use of reliable and validated assessment tools specifically adapted to this patient population. Early identification of malnourished patients enables the implementation of appropriate nutritional therapy and facilitates monitoring of treatment effectiveness [15,38].

In conclusion, the studies discussed here collectively highlight the multifaceted nature of nutritional assessment in oncology patients. The diversity of tools used, ranging from subjective assessments to objective measurements, underscores the need for a comprehensive and individualized approach, which can improve the precision of nutritional interventions, thereby benefiting clinical outcomes in patients diagnosed with cancer. The choice of a nutritional assessment tool should be guided by established recommendations, validation in independent studies conducted across diverse patient populations, and appropriate interpretation according to predefined diagnostic criteria.

## 5. Strengths and Limitations

This study has certain limitations that should be considered when interpreting its results. The small sample size may not be fully representative of the general population. We examined only oncology patients who were under medical care. These patients are more integrated into the healthcare system; therefore, the representativeness of the sample is limited. Conclusions should be drawn exclusively for this specific target group. Furthermore, the study focused on a population of oncology patients residing in Poland; therefore, the findings should not be generalized or assumed to be applicable to all oncology patients worldwide. The age groups were too broad, which meant that not all scales were adapted to the study group. This also distorted the final conclusions.

Despite these limitations, this study provides valuable information regarding the prevalence and burden of malnutrition in the oncology patient population. A strength of this study is that the nutritional status assessment was conducted within three days of hospital admission, allowing for early intervention. From this perspective, early screening and referral for treatment upon admission serve the essential purpose of improving the clinical prognosis regarding nutritional status through individualized intervention. This approach offers the potential to shorten hospital stays, reduce the risk of readmission, morbidity, and mortality, as well as improve treatment tolerance.

## 6. Conclusions

1. The prevalence of malnutrition among patients with cancer is high. Early nutritional screening and regular monitoring are essential for the timely identification of patients at risk of malnutrition. Understanding the scale of malnutrition among oncology patients is a crucial step toward its recognition and treatment;
2. Body weight deficits were significantly more frequently associated with tumor location, primarily lung as well as head and neck cancers;
3. All assessment tools utilized in this study can be applied to evaluate the risk of malnutrition. However, in preventive nutritional screening, priority should be given to instruments developed and validated for the specific patient population under assessment.

## Figures and Tables

**Table 1:** Descriptive statistics of the examined group of patients.

	Group I	Group II	Group III	Group IV	Group V
Demographic Information	Total N = 106	Total N = 120	Total N = 116	Total N = 108	Total N = 110
Characteristics % (N)					
Sex					
Women	83% (88)	58% (70)	49% (57)	44% (48)	59% (65)
Men	17% (18)	42% (50)	51% (59)	56% (60)	41% (45)
The age of the study group					
Standard deviations (SD)	59.51 (10.76)	55.15 (6.53)	53.55 (14.00)	51.23 (12.78)	55 (13.95)
Observed ranges	<19; 80>	<21; 69>	<29; 79>	<19; 70>	<24; 91>
Place of residence					
City	69% (73)	48% (58)	43% (50)	51% (55)	37% (41)
Village	31% (33)	52% (62)	57% (66)	49% (53)	63% (69)
Financial situation					
Very good	10% (11)	15% (18)	19% (22)	11% (12)	18% (20)
Good	42% (44)	34% (41)	53% (61)	23% (25)	33% (36)
Average	34% (36)	22% (26)	28% (33)	39% (42)	45% (50)
Bad	14% (15)	29% (35)	0% (0)	27% (29)	4% (4)
Age groups					
18–30	1% (1)	1% (1)	12% (14)	10% (9)	5% (5)
31–40	5% (5)	15% (18)	20% (23)	18% (22)	6% (7)
41–50	10% (11)	18% (22)	25% (29)	25% (27)	16% (18)
51–60	28% (30)	30% (36)	38% (44)	30% (32)	29% (32)
61–70	38% (40)	36% (43)	5% (6)	10% (11)	14% (15)
71–80	18% (19)	0% (0)	0% (0)	7% (7)	30% (33)
Education					
Primary	8% (8)	15% (18)	7% (8)	14% (15)	21% (23)
Vocational	35% (37)	26% (31)	25% (29)	44% (48)	23% (25)
Secondary	32% (34)	23% (28)	38% (44)	27% (29)	39% (43)
Higher	25% (27)	36% (43)	30% (35)	15% (16)	17% (19)
Marital status					
Married	68% (72)	57% (68)	60% (70)	42% (45)	51% (56)
Widowed	11% (12)	8% (10)	17% (20)	40% (43)	15% (17)
Unmarried	21% (22)	35% (42)	23% (26)	18% (20)	34% (37)
Professional activity					
Professionally active	37% (39)	19% (23)	61% (71)	53% (57)	28% (30)
Unemployed	5% (5)	10% (12)	2% (2)	4% (4)	15% (17)
Pension	4% (4)	41% (49)	17% (20)	19% (21)	30% (33)
Retirement	54% (58)	30% (36)	20% (23)	24% (26)	27% (30)

**Table 2:** Characteristics of cancer among the patients.

Variables	Group I	Group II	Group III	Group IV	Group V	*p*
Cancer stage						
Early-stage cancer	22% (23)	19% (23)	7% (8)	11% (12)	19% (21)	0.99
Intermediate stage cancer	24% (25)	37% (44)	46% (53)	40% (43)	21% (23)	0.21
Advanced-stage cancer	43% (46)	39% (47)	41% (48)	40% (43)	50% (55)	0.55
Very advanced-stage cancer	11% (12)	5% (6)	6% (7)	9% (10)	10% (11)	0.19
Treatment of cancer						
Surgical treatment	54% (57)	22% (26)	13% (15)	32% (34)	15% (16)	0.22
Chemotherapy	81% (86)	65% (78)	61% (71)	73% (79)	49% (54)	0.01
Hormonal treatment	7% (7)	5% (6)	0% (0)	3% (3)	10% (11)	0.15
Radiation therapy	22% (23)	31% (37)	26% (30)	19% (20)	15% (16)	0.09
Duration of cancer						
Up to 1 year	58% (62)	22% (26)	4% (5)	28% (30)	20% (22)	0.21
2–3 years	37% (39)	30% (36)	51% (59)	34% (37)	30% (33)	0.17
4–5 years	3% (3)	38% (46)	40% (46)	27% (29)	40% (44)	0.23
Over 5 years	2% (2)	10% (12)	5% (6)	11% (12)	10% (11)	0.23
Ailments						
Vomiting	46% (49)	51% (61)	28% (32)	10% (11)	67% (74)	0.01
Taste disturbances	12% (13)	8% (10)	25% (29)	16% (17)	11% (12)	0.19
Loss of appetite	59% (63)	58% (70)	23% (27)	54% (58)	71% (78)	0.01
Diarrhea	34% (36)	31% (37)	15% (17)	5% (5)	15% (16)	0.09
Persistent belching	31% (33)	15% (18)	9% (10)	5% (5)	21% (23)	0.18
Heartburn	31% (33)	33% (40)	57% (66)	2% (2)	25% (27)	0.21
Bloating	42% (45)	51% (61)	36% (42)	62% (67)	55% (60)	0.49
Constipation	47% (50)	55% (66)	26% (30)	13% (14)	61% (67)	0.15
Stomach pain	48% (51)	50% (60)	30% (35)	30% (32)	69% (76)	0.54
Stomatitis	49% (52)	54% (65)	55% (64)	25% (27)	62% (68)	0.01
Anosmia	10% (11)	80% (96)	23% (27)	15% (16)	31% (34)	0.01
Way of feeding						
Oral (natural nutrition)	79% (84)	97% (116)	77% (89)	79% (85)	82% (90)	0.22
Nasogastric tube (artificial nutrition)	8% (8)	0% (0)	2% (2)	4% (4)	8% (9)	0.19
PEG/PEJ (artificial nutrition)	1% (1)	1% (1)	19% (22)	15% (16)	6% (7)	0.09
Parenteral nutrition	12% (13)	2% (2)	2% (2)	2% (2)	4% (4)	0.15
Supplements	20% (21)	13% (16)	40% (46)	30% (32)	21% (23)	0.13
Industrial diet	13% (14)	44% (53)	32% (37)	33% (36)	52% (57)	0.55

**Table 3:** Nutritional status among the patients.

Variables	Group I	Group II	Group III	Group IV	Group V	*p*
BMI index						
Underweight	7% (7)	11% (13)	0% (0)	27% (29)	60% (66)	0.01
Normal	36% (38)	64% (77)	75% (87)	64% (69)	40% (44)	
Overweight	43% (46)	22% (26)	17% (20)	0% (0)	0% (0)	
Obesity	14% (15)	3% (4)	8% (9)	9% (10)	0% (0)	
Arm circumference MAC						
Below the gender norm	14% (15)	43% (52)	9% (10)	12% (13)	59% (65)	0.01
Above the gender norm	86% (91)	57% (68)	91% (106)	88% (95)	41% (45)	
Subjective assessment of nutritional status SGA						
Category A	20% (21)	21% (25)	54% (63)	21% (23)	23% (25)	0.15
Category B	67% (71)	50% (60)	28% (32)	37% (40)	45% (50)	
Category C	13% (14)	29% (35)	18% (21)	42% (45)	32% (35)	
Mini Nutritional Assessment MNA						
Proper nutritional status	14% (15)	11% (13)	0% (0)	0% (0)	0% (0)	0.01
Risk of malnutrition	72% (76)	45% (54)	58% (67)	9% (10)	20% (22)	
Malnutrition	14% (15)	44% (53)	42% (49)	91% (98)	80% (88)	
Nutritional Risk Screening Score 2002 NRS						
Nutritional therapy is indicated	43% (46)	47% (56)	58% (67)	55% (59)	80% (88)	0.01
conservative management	57% (60)	53% (64)	42% (49)	45% (49)	20% (22)	
Weight loss since diagnosis						
None	32% (34)	18% (21)	20% (23)	8% (8)	25% (27)	0.01
1–4 kg	38% (40)	24% (29)	56% (65)	22% (24)	55% (61)	
5–10 kg	25% (27)	49% (59)	21% (24)	45% (49)	19% (21)	
Over 10 kg	5% (5)	9% (11)	3% (4)	25% (27)	1% (1)	

**Table 4:** The value of the BMI index depending on the health situation of the all patients.

BMI Index	Underweight	Normal	Overweight	Obesity	*p*
Tumor location					
Group I	7% (7)	36% (38)	43% (46)	14% (15)	0.01
Group II	11% (13)	64% (77)	22% (26)	3% (4)	
Group III	0% (0)	75% (87)	17% (20)	8% (9)	
Group IV	27% (29)	64% (69)	0% (0)	9% (10)	
Group V	60% (66)	40% (44)	0% (0)	0% (0)	
Treatment time					
Up to 1 year	15% (22)	56% (81)	21% (30)	8% (12)	0.40
2–3 years	8% (16)	69% (141)	21% (43)	2% (4)	
4–5 years	40% (67)	40% (67)	20% (34)	0% (0)	
over 5 years	35% (15)	47% (20)	18% (8)	0% (0)	
Level of advancement					
Early-stage cancer	13% (11)	43% (37)	35% (31)	9% (8)	0.01
Intermediate stage cancer	0% (0)	72% (135)	16% (30)	12% (23)	
Advanced-stage cancer	17% (41)	63% (150)	17% (41)	3% (7)	
Very advanced-stage cancer	25% (11)	58% (27)	17% (8)	0% (0)	
The way of nutrition					
Natural nutrition	14% (65)	56% (260)	23% (107)	7% (32)	0.01
Artificial nutrition	9% (17)	77% (143)	14% (26)	0% (0)	
Sex					
Women	10% (33)	63% (207)	19% (62)	8% (26)	0.35
Men	18% (42)	57% (132)	23% (53)	2% (5)	
Place of residence					
City	13% (36)	59% (163)	26% (72)	2% (6)	0.61
Village	13% (37)	61% (173)	18% (51)	8% (22)	
Age groups					
≤50	10% (21)	73% (155)	15% (32)	2% (4)	0.01
51–70	13% (38)	56% (162)	22% (63)	9% (26)	
>71	21% (12)	42% (25)	32% (19)	5% (3)	
Marital status					
Married	18% (56)	57% (177)	18% (56)	7% (22)	0.10
Widowed	14% (14)	48% (49)	31% (32)	7% (7)	
Unmarried	0% (0)	86% (126)	14% (21)	0% (0)	
Education					
Primary	9% (6)	59% (43)	23% (17)	9% (6)	0.82
Vocational	17% (29)	58% (99)	25% (42)	0% (0)	
Secondary	17% (30)	61% (109)	17% (30)	5% (9)	
Higher	5% (7)	63% (88)	21% (29)	11% (16)	
Financial situation					
Very good	11% (9)	68% (57)	16% (13)	5% (4)	0.98
Good	11% (23)	60% (124)	20% (41)	9% (19)	
Average, bad	15% (41)	58% (156)	23% (62)	4% (11)	

**Table 5:** The value MNA depending on the health situation of the all patients.

MNA	Proper Nutritional Status	Risk of Malnutrition	Malnutrition	*p*
Tumor location				
Group I	14% (15)	72% (76)	14% (15)	0.01
Group II	11% (13)	45% (54)	44% (53)	
Group III	0% (0)	58% (67)	42% (49)	
Group IV	0% (0)	9% (10)	91% (98)	
Group V	0% (0)	20% (22)	80% (88)	
Treatment time				
Up to 1 year	2% (3)	56% (81)	42% (61)	0.01
2–3 years	15% (31)	31% (63)	54% (110)	
4–5 years	40% (67)	20% (34)	40% (67)	
over 5 years	58% (25)	28% (12)	14% (6)	
Level of advancement				
Early-stage cancer	30% (26)	57% (50)	13% (11)	0.01
Intermediate stage cancer	8% (15)	56% (105)	36% (68)	
Advanced-stage cancer	0% (0)	41% (98)	59% (141)	
Very advanced-stage cancer	0% (0)	17% (8)	83% (38)	
The way of nutrition				
Natural nutrition	10% (46)	48% (223)	42% (195)	0.09
Artificial nutrition	1% (2)	35% (65)	64% (119)	
Sex				
Women	3% (10)	53% (174)	44% (144)	0.02
Men	16% (37)	34% (79)	50% (116)	
Place of residence				
City	18% (50)	46% (127)	36% (100)	0.01
Village	3% (9)	45% (127)	52% (147)	
Age groups				
≤50	12% (26)	44% (93)	44% (93)	0.65
51–70	4% (12)	50% (144)	46% (133)	
>71	10% (6)	37% (22)	53% (31)	
Marital status				
Married	3% (9)	43% (134)	54% (168)	0.01
Widowed	7% (7)	52% (53)	41% (42)	
Unmarried	24% (35)	43% (63)	33% (49)	
Education				
Primary	32% (23)	27% (19)	41% (30)	0.01
Vocational	0% (0)	54% (92)	46% (78)	
Secondary	5% (9)	39% (69)	56% (100)	
Higher	0% (0)	68% (95)	32% (45)	
Financial situation				
Very good	11% (9)	47% (39)	42% (35)	0.01
Good	20% (41)	49% (102)	31% (64)	
Average, bad	0% (0)	42% (113)	58% (157)	

**Table 6:** The value NRS depending on the health situation of the all patients.

NRS	Nutritional Treatment Indicated	Conservative Treatment	*p*
Tumor location			
Group I	43% (46)	57% (60)	0.46
Group II	47% (56)	53% (64)	
Group III	58% (67)	42% (49)	
Group IV	55% (59)	45% (49)	
Group V	80% (88)	20% (22)	
Treatment time			
Up to 1 year	45% (65)	55% (80)	0.51
2–3 years	54% (110)	46% (94)	
4–5 years	80% (134)	20% (34)	
Over 5 years	58% (25)	42% (18)	
Level of advancement			
Early-stage cancer	22% (19)	78% (68)	0.01
Intermediate stage cancer	52% (98)	48% (90)	
Advanced-stage cancer	57% (136)	43% (103)	
Very advanced-stage cancer	75% (34)	25% (12)	
The way of nutrition			
Natural nutrition	46% (213)	54% (251)	0.15
Artificial nutrition	64% (119)	36% (67)	
Sex			
Women	44% (144)	56% (184)	0.11
Men	59% (137)	41% (95)	
Place of residence			
City	44% (122)	56% (155)	0.31
Village	54% (153)	46% (130)	
Age groups			
≤50	34% (72)	66% (140)	0.01
51–70	51% (145)	50% (144)	
>71	84% (50)	16% (9)	
Marital status			
Married	54% (168)	46% (143)	0.23
Widowed	55% (56)	45% (46)	
Unmarried	33% (49)	67% (98)	
Education			
Primary	55% (40)	45% (32)	0.32
Vocational	58% (99)	42% (71)	
Secondary	51% (91)	49% (87)	
Higher	32% (45)	68% (95)	
Financial situation			
Very good	37% (31)	63% (52)	0.32
Good	40% (83)	60% (124)	
Average, bad	62% (167)	38% (103)	

**Table 7:** The value SGA depending on the health situation of the all patients.

SGA	Category A	Category B	Category C	*p*
Tumor location				
Group I	20% (21)	67% (71)	13% (14)	0.01
Group II	21% (25)	50% (60)	29% (35)	
Group III	54% (63)	28% (32)	18% (21)	
Group IV	21% (23)	37% (40)	42% (45)	
Group V	23% (25)	45% (50)	32% (35)	
Treatment time				
Up to 1 year	12% (17)	46% (67)	42% (61)	0.01
2–3 years	15% (31)	45% (92)	40% (81)	
4–5 years	26% (44)	20% (33)	54% (91)	
over 5 years	58% (25)	38% (16)	4% (2)	
Level of advancement				
Early-stage cancer	30% (26)	57% (50)	13% (11)	0.01
Intermediate stage cancer	10% (19)	56% (105)	34% (64)	
Advanced-stage cancer	1% (2)	40% (96)	59% (141)	
Very advanced-stage cancer	7% (3)	13% (6)	80% (37)	
The way of nutrition				
Natural nutrition	9% (42)	48% (223)	43% (199)	0.01
Artificial nutrition	6% (11)	30% (56)	64% (119)	
Sex				
Women	23% (75)	39% (128)	38% (125)	0.25
Men	22% (51)	53% (123)	25% (58)	
Place of residence				
City	20% (55)	44% (122)	36% (100)	0.19
Village	13% (37)	35% (99)	52% (147)	
Age groups				
≤50	22% (47)	34% (72)	44% (93)	0.55
51–70	14% (40)	40% (116)	46% (133)	
>71	17% (10)	30% (18)	53% (31)	
Marital status				
Married	13% (40)	34% (106)	53% (165)	0.51
Widowed	10% (10)	50% (51)	40% (41)	
Unmarried	27% (40)	40% (59)	33% (48)	
Education				
Primary	12% (8)	37% (27)	51% (37)	0.01
Vocational	10% (17)	44% (75)	46% (78)	
Secondary	10% (18)	58% (103)	32% (57)	
Higher	5% (7)	39% (55)	56% (78)	
Financial situation				
Very good	18% (15)	40% (33)	42% (35)	0.21
Good	29% (60)	40% (83)	31% (64)	
Average, bad	10% (27)	32% (86)	58% (157)	

## Data Availability

The original contributions presented in this study are included in the article. Further inquiries can be directed to the corresponding author.
